# Feature saliency and feedback information interactively impact visual category learning

**DOI:** 10.3389/fpsyg.2015.00074

**Published:** 2015-02-19

**Authors:** Rubi Hammer, Vladimir Sloutsky, Kalanit Grill-Spector

**Affiliations:** ^1^Department of Psychology, Stanford UniversityStanford, CA, USA; ^2^Department of Communication Sciences and Disorders, Northwestern UniversityEvanston, IL, USA; ^3^Interdepartmental Neuroscience Program, Northwestern UniversityEvanston, IL, USA; ^4^Department of Psychology and Center for Cognitive Science, The Ohio State UniversityColumbus, OH, USA; ^5^Stanford Neuroscience Institute, Stanford UniversityStanford, CA, USA

**Keywords:** category learning, categorization, attentional learning, perceptual learning, visual perception, feedback processing, feature saliency, perceptual expertise

## Abstract

Visual category learning (VCL) involves detecting which features are most relevant for categorization. VCL relies on attentional learning, which enables effectively redirecting attention to object’s features most relevant for categorization, while ‘filtering out’ irrelevant features. When features relevant for categorization are not salient, VCL relies also on perceptual learning, which enables becoming more sensitive to subtle yet important differences between objects. Little is known about how attentional learning and perceptual learning interact when VCL relies on both processes at the same time. Here we tested this interaction. Participants performed VCL tasks in which they learned to categorize novel stimuli by detecting the feature dimension relevant for categorization. Tasks varied both in feature saliency (low-saliency tasks that required perceptual learning vs. high-saliency tasks), and in feedback information (tasks with mid-information, moderately ambiguous feedback that increased attentional load, vs. tasks with high-information non-ambiguous feedback). We found that mid-information and high-information feedback were similarly effective for VCL in high-saliency tasks. This suggests that an increased attentional load, associated with the processing of moderately ambiguous feedback, has little effect on VCL when features are salient. In low-saliency tasks, VCL relied on slower perceptual learning; but when the feedback was highly informative participants were able to ultimately attain the same performance as during the high-saliency VCL tasks. However, VCL was significantly compromised in the low-saliency mid-information feedback task. We suggest that such low-saliency mid-information learning scenarios are characterized by a ‘cognitive loop paradox’ where two interdependent learning processes have to take place simultaneously.

## INTRODUCTION

The human brain is capable of managing effectively an immense amount of visual information, rendering it rapidly into a reliable and meaningful representation of objects and events. The cognitive process enabling this is Visual Category Learning (VCL), which involves the detection of object features that are most relevant for categorization. In many learning scenarios VCL requires supervision that may involve the processing of labeled exemplars (Lupyan et al., 2007; Davis et al., 2012; but see also Sloutsky et al., 2007), or being informed that few objects are from the same-category or from different categories without the use of labels (Namy and Gentner, 2002; Hammer et al., 2009a,b; Mathy et al., 2013). Deducing associations between category labels and exemplars, or the categorical relation between few objects, can be accomplished by feedback that follows the learner decision, indicating whether the decision was correct or not (Love, 2002; Maddox et al., 2003; Daniel and Pollmann, 2010; Antzoulatos and Miller, 2011; Lopez-Paniagua and Seger, 2011).

In everyday life scenarios, information provided by feedback during VCL may often be suboptimal, or even misleading. Thus, multiple learning experiences are required to ultimately enable categorizing objects based only on those attributes that are most relevant for categorization (Hammer et al., 2007, 2008). Another challenge characterizing some VCL scenarios is low-saliency and poor representation of features that are relevant for categorization, which may result in such features being overlooked. Facing these challenges, VCL relies on two fundamental processes. The first process is attentional learning, which enables the volitional allocation of attention to relevant features, while ‘filtering out’ distracting salient features that have little relevance for categorization (Rehder and Hoffman, 2005; Sloutsky and Fisher, 2008; Blair et al., 2009; Hoffman and Rehder, 2010; Sloutsky, 2010; McColeman et al., 2014). The second process is perceptual learning, which enables becoming more sensitive to subtle, initially hard to detect differences between objects from different categories (Shiffrin and Schneider, 1977; Goldstone, 1994, 1998; Goldstone et al., 2001; Roelfsema et al., 2010). Although previous studies showed that both attentional learning and perceptual learning contribute to the reduction of categorization errors, to-date no study systematically tested VCL tasks in which these two processes have to take place simultaneously.

Attentional learning involves improving the ability to allocate attention to important sensory information, within a given context, while ignoring task-irrelevant information (Nosofsky, 1984; Nosofsky and Palmeri, 1996; Kruschke and Blair, 2000; Sloutsky and Fisher, 2008; Smith et al., 2010). This often results in a rivalry between bottom-up processes where salient features attract more attention than less salient ones (Itti et al., 1998), and top-down processes driven by prior knowledge and expectations regarding which stimuli features have been found to be important in the past (Koch and Tsuchiya, 2007; Baluch and Itti, 2011; but see also Awh et al., 2012). These two attention processes are likely based on two primary brain networks: (i) The ventral attention network acts as a bottom-up saliency detection system enabling the involuntarily reorientation of attention to unexpected salient stimuli in the environment (Corbetta et al., 2008; Vossel et al., 2014). (ii) The dorsal attention network enables short-term circulation of information that is with current subjective importance, and top-down volitional direction of attention to chosen stimulus features (LaBar et al., 1999; Awh and Jonides, 2001). The nature of the interaction between these two attention brain networks is not fully understood. However, this interaction is thought to depend on context, the maturation of prefrontal executive brain regions, and subjective experiences (Gazzaley and Nobre, 2012; Weissman and Prado, 2012; Hammer et al., submitted).

Perceptual learning often results in increased sensitivity and long-lasting improvement in the ability to respond to previously undetected or poorly represented features (Ahissar and Hochstein, 1997; Goldstone, 1998; Fahle and Poggio, 2002). There is an ongoing debate regarding the role of top-down attention control in mediating visual perceptual learning. Presently, most behavioral studies suggest that effective perceptual learning requires intentional direction of attention to the features being learned (Ahissar and Hochstein, 1993; Schoups et al., 2001). Such attentional control may be mediated by corrective feedback that enables the perceiver to realize that two stimuli that were initially confused as being the same are in fact different. In turn, this process may trigger attentional search for visual features that enables differentiating between objects and object categories, eventually resulting in an increased sensitivity to these features (Herzog and Fahle, 2002; Aberg and Herzog, 2012). Others suggest that in some scenarios stimulus-reward pairing may result in “task-irrelevant” perceptual learning, or unintended changes in stimuli representation that may affect later behavior. This may happen when an ‘unattended task-irrelevant’ sensory feature is being associated with a rewarding outcome (Seitz et al., 2009; Roelfsema et al., 2010).

The *perceived* feature saliency, and thus the degree to which an object attribute attracts attention, may be altered due to changes in representation such as those that follow perceptual learning. On the other hand, perceptual learning may require effective top-down attention control enabling prolonged focusing of attention to a specific feature (Goldstone and Steyvers, 2001; Serences and Yantis, 2006; Seitz et al., 2009). Arguably, when the objects of interest are complex and differ in multiple low-saliency features, VCL is less likely to be effective without informative guidance (Hammer, submitted). These suggest that perceptual learning and attentional learning do not only affect VCL independently, but they may have a complex context-dependent interaction, where the two learning processes rely on one another and cannot take place at the same time, effectively. Here we tested this interaction by systematically manipulating feature saliency and feedback information. We expected that low-saliency of features relevant for categorization would increase the reliance of VCL on perceptual learning. We expected that ambiguous feedback would hinder VCL by reducing the odds that attention would be exclusively directed to the task-relevant feature dimension in successive learning trials. Consequently, VCL tasks with low-saliency features and ambiguous feedback would rely both on perceptual learning and attentional learning, where the two processes have to take place simultaneously. Such scenarios, where two interdependent learning processes have to take place simultaneously, may involve a ‘cognitive loop paradox’ with a distinct negative impact on VCL.

We define feature saliency in terms of the physical dissimilarity between objects along a given feature dimension, in a given context (Diesendruck et al., 2003; Hammer and Diesendruck, 2005; Chen et al., 2013). For example, when categorizing Dobermans (large dogs) and Chihuahuas (small dogs), body-size is a high-saliency feature dimension due to salient dissimilarities in body size between these two categories of dogs. On the other hand, when categorizing Labradors and Labradoodles (both are mid-large size dogs), body-size is a low-saliency feature dimension due to high similarities in body size between the two categories of dogs. Note that when two stimuli are perceived as substantially dissimilar from one another across a particular feature dimension, this feature dimension may attract more attention and may *a-priori* be considered as having higher diagnostic value than a low-saliency feature dimension (Rosch and Mervis, 1975; Tversky, 1977; Nosofsky, 1986; Kruschke, 2003; Chin-Parker and Ross, 2004).

In the current study, in each VCL task stimuli differed from one another in three feature dimensions (e.g. the body width, limbs shape and horns thickness of novel creature-like stimuli) where only one feature dimension was relevant for categorization. In each of the VCL tasks we kept feature saliency similar across the three feature dimensions, making them similarly likely to be *a-priori* perceived as relevant for categorization. We contrasted between high-saliency VCL tasks and low-saliency VCL tasks, investigating how feedback information is being used in these two learning scenarios. High-saliency tasks simulate scenarios where the categorized objects may differ in several salient attributes, only some of which are important for categorization. Low-saliency tasks simulate scenarios where a greater degree of perceptual expertise is required for detecting fine yet important differences between objects. We argue that in this later scenario, introducing highly informative feedback would be critical for VCL to be effective.

**FIGURE 1:**
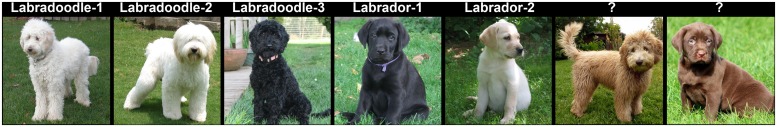
**Learning to categorize puppies by comparing and contrasting paired-examples.** The most salient feature dimensions differentiating the seven puppies are fur color and fur length. Being informed that two puppies are from the same category can be useful for detecting which feature dimension is most important for categorization. For example, comparing Labradoodle-3 with Labradoodle-1 or Labradoodle-2, or Labrador-1 with Labrador-2, while being informed about their categorical relation, is informative for learning that salient differences in fur color are not important for categorizing these dogs, and thus fur length is more likely to be important. On the other hand, comparing Labradoodle-1 with Labradoodle-2 does not enable inferring which feature is most important. Being informed that two puppies are from distinct categories is also useful forVCL, enabling detecting differences between categories. For example, comparing Labradoodle-3 with Labrador-1, or Labrador-2 with Labradoodle-1 or Labradoodle-2, is informative by highlighting the between categories differences in fur length. On the other hand, comparing Labradoodle-3 with Labrador-2, or Labrador-1 with Labradoodle-1 or Labradoodle-2, is less informative since such comparison is less constraining, enabling deducing that fur color and/or fur length are important for categorization.The labeled examples allow learning that fur length is most relevant for categorization, which in turn allows properly categorizing the two rightmost puppies despite their distinct color(see (see Hammeret al., 2008 for a formal discussion).

We determined the quantity of feedback information as the degree of ambiguity in each learning trial, where ambiguous feedback did not provide all the information needed for decisive detection of the task-relevant feature dimension in a single learning trial. Ambiguous feedback should not be confused with an inherently incorrect or misleading feedback. Here, in each trial two stimuli were presented simultaneously and the participant had to decide if the two were from the same-category or from different-categories. In high-information learning trials, same-category pairs were identical in the task-relevant feature dimension and differed in the two irrelevant feature dimensions, whereas different-categories pairs were different only in the task-relevant feature dimension and were identical in the two irrelevant feature dimensions. This enabled detecting the task-relevant feature dimension using the feedback from a single trial. In mid-information learning trials same-category stimuli were identical in the task-relevant feature dimension but also in one of the irrelevant feature dimensions. In different-categories trials the two stimuli were different in the task-relevant feature dimension and one of the irrelevant feature dimensions. Across multiple trials, the objective category relation between paired stimuli (as it could be deduced from the feedback) was not consistently associated with the irrelevant feature dimensions, but was consistently associated with the relevant feature dimension. Thus, inferring the relevant feature dimension (the categorization rule) was feasible but required integrating information across more trials. These principles are illustrated in **Figure 1**.

We hypothesized that ambiguous feedback would significantly compromise VCL in low-saliency conditions. In such conditions perceptual learning is more likely to be required not only for increasing sensitivity to important between categories differences, but also for improving the capacity to detect such fine differences, to begin with. In low-saliency VCL conditions features relevant for categorization are not well represented in sensory cortices and thus are likely to be left unattended during early learning trials, specifically when other features may initially be perceived as similarly important (Nosofsky and Palmeri, 1996; Goldstone, 1998; Goldstone et al., 2001). We expected that in such low-saliency conditions ambiguity in feedback would result in prolonged and extensive effort for detecting and isolating task-relevant feature dimensions, since this has to take place simultaneously with becoming more sensitive to differences between stimuli in these feature dimensions (Treisman and Gelade, 1980; Borji and Itti, 2013). In fact, frequently switching attention between several low-saliency feature-dimensions across learning trails, due to ambiguous feedback, can in some extreme scenarios utterly block perceptual learning. Such frequent attention switching may hinder the buildup of brain activation associated with the task-relevant features, preventing it from reaching the threshold required for significant changes in representation to take place (Goldstone, 1994; Gilbert et al., 2001; Kruschke, 2001).

On the other hand, we hypothesized that salient differences between stimuli would be easily detected. In such conditions it is possible to value the importance of each feature dimension by systematic volitional switching of attention between feature dimensions, even when the feedback is ambiguous. It is important to clarify that we expect ambiguous feedback to increase attentional load regardless of features saliency; but, we suggest that when features relevant for categorization are with high-saliency, and the feedback is not too ambiguous, such an increase in attentional load would not be necessarily evident in compromised VCL performances, or would have only small negative effect on VCL. When a feature dimension differentiating between categories would be with low-saliency yet the feedback would be informative, attention could be effectively directed to this feature dimension in multiple successive learning trials, enabling effective perceptual learning. We suggest that low-feature saliency is always expected to increase the dependency on perceptual learning, and thus would always inhibit VCL. Nevertheless, when the available feedback is informative, VCL would become reasonably manageable even under some low-saliency conditions.

## MATERIALS AND METHODS

### PARTICIPANTS

Sixty paid adults (36 females), with normal or corrected to normal vision, participated in the experiment. Participants gave informed written consent in accordance with a protocol approved by the Stanford University Institutional Review Board.

### EQUIPMENT AND SETTING

Psychtoolbox (MATLAB^®^) was used for stimuli presentation on a 1920 × 1200 pixels computer display and for the recording of participants’ responses. Participants’ heads were located about 70 cm (∼2 feet) from the computer screen such that each one of the two simultaneously presented stimuli occupied approximately 14° of the visual field.

### STIMULI

**FIGURE 2:**
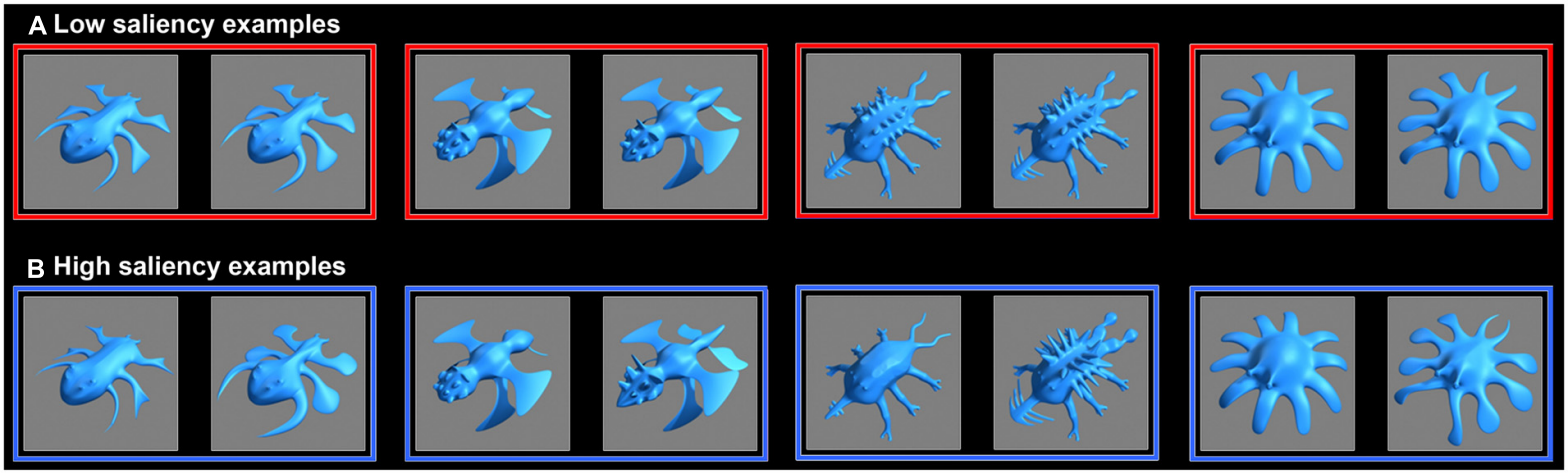
**Stimuli examples.** Low-saliency pairs **(A)** and corresponding high-saliency pairs **(B)** from each one of the four stimuli sets. Each paired stimuli differ in all three varying feature dimensions (see also Appendix A in the online supplemental).

We used four distinct sets of novel creature-like stimuli. In each set stimuli varied in three feature dimensions, produced from one standard object and three morph-target objects. Each morph-target differed from the standard in one feature dimension (e.g., shape of head or limb size). Stimuli were generated by morphing between the standard object and each of the three morph-targets using morphing values with steps of 11% (0% to 99%; total of 10 morph levels within each feature dimension). For the experimental VCL tasks, from each stimuli set eight stimuli were used in each saliency-condition so that stimuli differed in three binary feature dimensions. In VCL tasks with high-feature saliency the selected stimuli differed from one another in high morph values within each feature dimension (77% or higher). In low-saliency VCL tasks stimuli differed in substantially lower morph values (33% or lower). The selection of stimuli to be used in the experiment was determined based on pilot tests assuring that within each stimuli-set differences in the three feature dimensions were similarly likely to be detected when contrasting between paired stimuli that differed in a single feature dimension. Low-saliency differences were initially less likely to be detected (see Results for pre-learning performances). Due to the nature of stimuli (complex 3D renderings), fully equalizing feature saliency across the three feature dimensions and across all stimuli sets was not practicable, but the use of four stimuli sets and the counterbalancing across conditions insured that this would not have significant impact on the results (**Figure 2**; Appendix A in the online supplemental).

In each VCL task there were two predetermined categories. Stimuli in each category had an identical value on one feature dimension (the diagnostic task-relevant feature dimension) and differed in the other two (irrelevant) feature dimensions.

### DESIGN

Experimental conditions differed in feature saliency (high-saliency vs. low-saliency) and two primary levels of feedback information (high-information vs. mid-information). In addition to these four experimental conditions, we tested participants in two types of control tasks: No-information tasks where feedback was provided but it was not informative, and unsupervised categorization tasks with no feedback. These allowed assessment of the contribution of feedback information to VCL in the primary experimental conditions. The control VCL tasks also varied in levels of feature saliency.

Each participant performed four VCL tasks in four pseudo-randomly selected conditions (out of four possible experimental conditions and four possible control conditions). For each participant, each VCL task was based on a different stimulus set, and thus the participant had no prior experience with the stimuli at the beginning of each task. Tasks were counterbalanced so that each one of the four stimuli sets was used a similar number of times in each one of the four experimental conditions and four control conditions.

**FIGURE 3:**
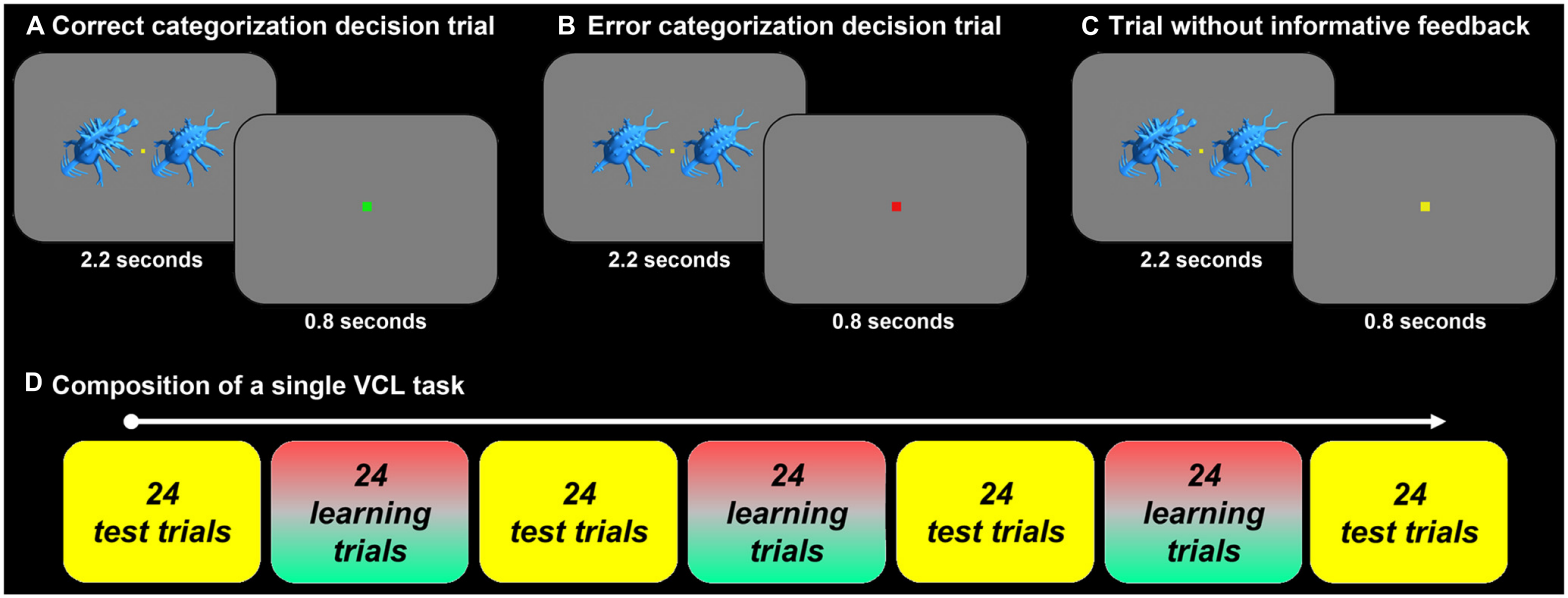
**(A)** An illustration of a learning trial with feedback indicating correct categorization decision (signified to the participant by a small green square presented after the execution of the categorization decision and stimuli offset). In each trial paired stimuli were presented for 2.2 seconds, during which the participant had to decide if the two creatures were from the same-category or different-categories. Feedback was presented during the following 0.8 seconds. **(B)** An illustration of a learning trial with feedback indicating incorrect categorization decision (signified by a small red square). **(C)** An illustration of a trial without informative feedback (a small yellow square signified an on-time response). Such trials were in the test-blocks and in the ‘learning-blocks’ in the unsupervised categorization control tasks. **(D)** Schematics of the composition of a single VCL task. Each VCL tasks lasted 8.5 minutes and included four test-blocks that alternated with three learning-blocks. In the unsupervised categorization tasks there were only seven test blocks. Each block included 24 trials.

Each VCL task included seven blocks of 24 trials each, where four test-blocks (T1, T2, T3, T4) alternated with three learning-blocks (L1, L2, L3). In each trial two creatures were presented simultaneously for 2.2 seconds during which the participant had to decide if the two belong to the same category or to different categories by pressing one of two keys. This was followed by 0.8 seconds inter-trial interval during which the feedback was presented. In learning-blocks in the high-information and mid-information experimental conditions, and the no-information control condition, a green square indicated a correct categorization decision and a red square an incorrect/error decision. In the unsupervised ‘learning-blocks,’ and in the test-blocks, a yellow square indicated an on-time response (**Figure 3**).

Participants were instructed that in each VCL task they have to categorize the creatures into two distinct subspecies based on one critical attribute (the task-relevant feature dimension), which can be inferred from the feedback. Participants were told that if they find the feedback to be ineffective, they should guess which feature dimension is most likely to be task-relevant. Participants were told that two creatures of the same subspecies should be identical in this feature dimension, and two creatures from distinct subspecies should differ in this feature dimension. Prior to performing the experimental tasks, the participant performed short warm-up tasks simulating the experimental conditions she was expected to perform (using dedicated stimuli sets that were not used in the experimental tasks). This allowed familiarizing participants with the experimental setting (but not with the stimuli used for the experimental tasks). The duration of each VCL task was 8.5 minutes, with a few minutes break between tasks. The overall duration of an experimental session was 60–75 minutes.

**FIGURE 4:**
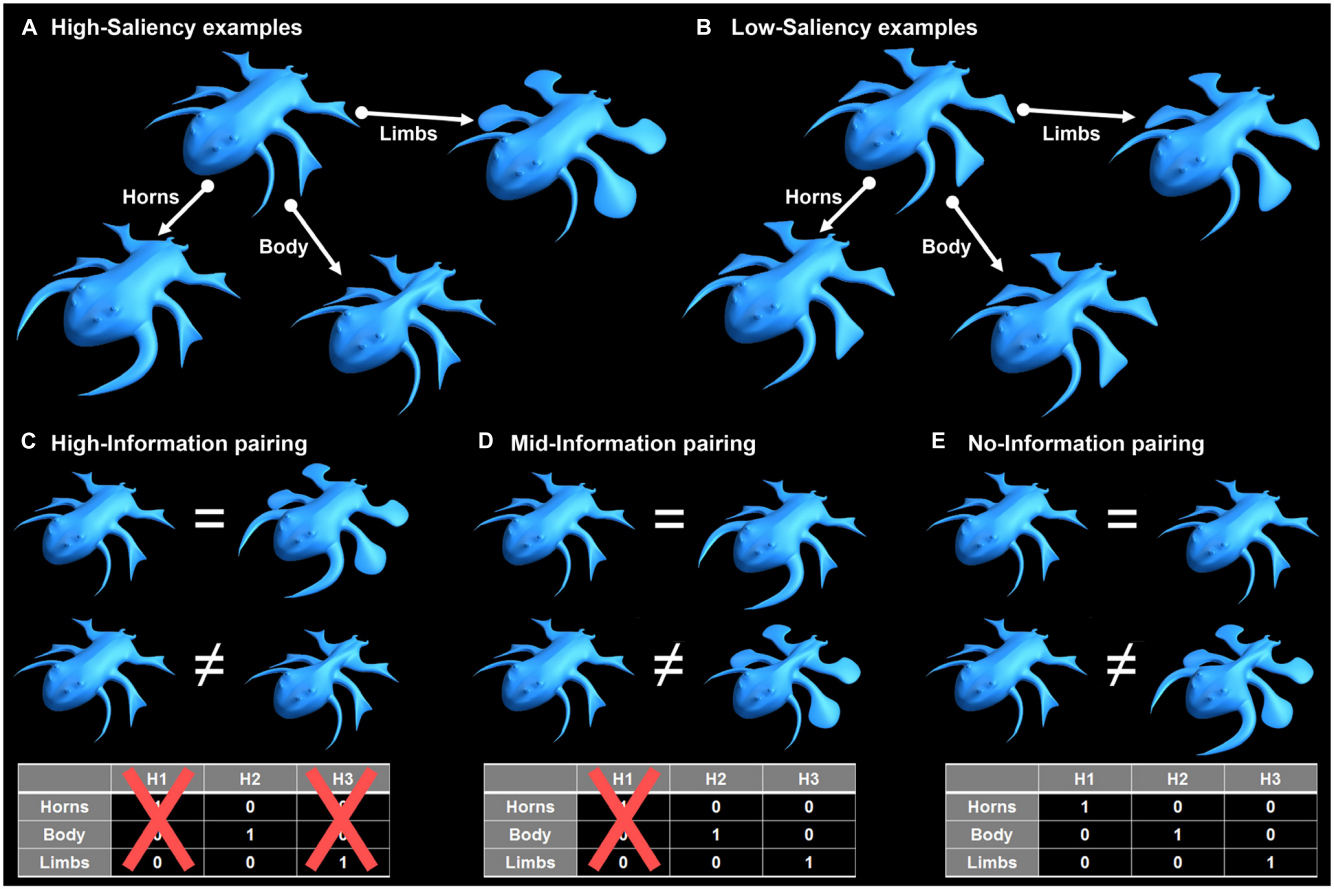
**Creature-pairing compositions.** **(A)** Example of high-saliency feature dimensions. **(B)** Example of low-saliency feature dimensions. **(C)** Examples of same-category (upper) and different-categories (lower) high-information pairs (using the high-saliency stimuli). The portion of feature dimensions detected as relevant based on a given trial determined the quantity of information provided in this trial. Here, the only feasible ‘hypothesis’ that could be considered by the participant was that the creatures’ body width is relevant for categorization (-log _2_1/3 = 1.585 bits). **(D)** Examples of same-category (upper) and different-categories (lower) mid-information pairs. Based on these examples, either the creatures’ body width or their limbs could be relevant for categorization (-log _2_2/3 = 0.585 bits). **(E)** Examples of same-category (upper) and different-categories (lower) pairs with no-information (control task). Based on these examples, the creatures’ body width, their limbs or their horns could be relevant for categorization (-log _2_3/3 = 0 bits). In learning-blocks, the category relation between the paired stimuli could be deduced from the feedback that followed a categorization decision. The tables at the bottom describe the possible hypothesis space. When all feature dimensions are salient and participants are informed that only one feature dimension is task-relevant, VCL requires the participant deciding which of the 3 hypotheses (H1–H3) is correct. When features are not salient, and the participant is more likely to be unaware of any between-stimuli differences, VCL require the participant rejecting the null hypothesis stating, “all creatures are the same”.

In high-saliency VCL tasks both the within-category and between-categories differences were with high-saliency (**Figure 4A**), and in low-saliency tasks both the within-category and between-categories differences were with low-saliency (**Figure 4B**).

In learning trials with high-information feedback, paired same-category creatures were identical in the relevant feature dimension and differed in the two irrelevant feature dimensions (upper pair in **Figure 4C**). Paired different-categories creatures differed in the relevant feature dimension and were identical in the two irrelevant ones (lower pair in **Figure 4C**). That is, each high-information learning trial either indicated all the within-category variability (same-category pairs), or pinpointed the diagnostic feature dimension differentiating between-categories (different-categories pairs). High-information learning trials can be also formulated as trials in which the task-relevant feature dimension and the irrelevant feature dimensions were anti-correlated (correlation of -1): Where ‘*A*’ denotes the relevant feature dimension, ‘*B*’ and ‘*C*’ the irrelevant feature dimensions and ‘*X*’ the categorization decision outcome, the only possible inferred trial-by-trial causality in the high-information condition was (four representative trials): (A → X) ∩ (A → X) ∩ (A → X) ∩ (A → X)⋯

In the learning trials with mid-information feedback, paired same-category creatures were identical in the relevant feature dimension and one of the two irrelevant feature dimensions (randomly alternating between the two across different trials; upper pair in **Figure 4D**). Paired different-categories creatures differed in the relevant feature dimension and one of the irrelevant feature dimensions (again, alternating between the two across trials; lower pair in **Figure 4D**). That is, in each trial there was a degree of ambiguity regarding which feature dimension is relevant for categorization, forcing the use of information from several trials in order to confidently learn the categorization rule. Mid-information learning trials can be also formulated as trials in which there was partial correlation between the task-relevant feature dimension and each one of the two irrelevant feature dimensions: (A ∪ B → X) ∩ (A ∪ C → X) ∩ (A ∪ C → X) ∩ (A ∪ B → X)⋯

In the learning trials with no-information feedback, paired same-category creatures were identical in all feature dimensions (upper pair in **Figure 4E**), whereas paired different-categories creatures differed in all feature dimensions (lower pair in **Figure 4E**). Here the degree of ambiguity was such that the categorization rule could not be inferred even by integrating information across an infinite number of trials. No-information learning trials can be formulated as trials in which the correlation between the task-relevant feature dimension and the two irrelevant feature dimensions is maximized (+1). In such scenarios decisively inferring that a single feature dimension is relevant for categorization was impossible: (A∪B ∪C → X) ∩ (A∪B ∪C → X) ∩ (A∪B ∪C → X) ∩ (A∪B ∪C → X)⋯ (see Schulz et al., 2007 and Shafto et al., 2008 for a similar formulation of ambiguity in inductiontasks).

In the unsupervised ‘learning-blocks’ composition of paired stimuli was the same as in the test blocks, where paired stimuli always differed in two feature dimensions. In each test-block trial, paired creatures always differed in two feature dimensions. Same-category pairs differed in the two irrelevant feature dimensions and were identical in the relevant one (such as in the upper pair in **Figure 4C**). Different-categories pairs differed in the relevant feature dimension and in one of the irrelevant feature dimensions, and were identical in the other irrelevant feature dimension (such as in the lower pair in **Figure 4D**). This prevented participants from making same/different categorization decision based on overall similarity between stimuli, and it ensured that all pair-wise correlations between the three feature dimensions are identical (the relevant feature dimension could not be inferred from the stimuli presentation statistics).

### PERFORMANCE MEASUREMENTS

We define a “Hit” as correctly identifying two creatures as members of the same-category, and a “False-Alarm” as incorrectly identifying two creatures of different-categories as members of the same-category. Based on the Hit and False-Alarm rate we calculated the participant’s accuracy using the non-parametric measure A-prime (Grier, 1971; Stanislaw and Todorov, 1999). A-prime = 0.5 indicates chance-level performance and A-prime = 1.0 indicates perfect performance. A-prime scores close to 0.0 indicate high sensitivity to category identity but with consistently reversed responses (i.e., categorizing items from two categories as belonging to the same category, and categorizing items from the same category as belonging to different categories). Hit rate and False-Alarm rate are defined as:

$$H=Hit\,\;\;rate=\frac{Hits}{Hits+Misses}$$

F=False⁢  Alarm⁢ rate=False⁢  AlarmsFalse⁢  Alarms+Correct⁢  Rejections

A-prime is defined as:

$$A\prime =0.5+sign\left(H-F\right)\times \frac{{\left(H-F\right)}^{2}+|H-F|}{4\times \max(H,F)-4\times H\times F\;}$$

### KEY PERFORMANCE BENCHMARKS

Participants’ learning capabilities were evaluated based on several benchmarks accounting for the constraints of stimuli pairing in learning and test blocks, and the correlations between feature dimensions: making random same/different decisions would yield chance performance, A-prime = 0.5; categorizing based on the relevant feature dimension would yield perfect performance, A-prime = 1; systematically referring to an irrelevant feature dimension during a test block or during an unsupervised ‘learning-block’ would yield an A-prime = 0.12 (with Hit rate = 0 and False-Alarm rate = 0.5); systematically referring to an irrelevant feature dimension during a learning block with mid-information feedback would yield an A-prime = 0.5; due to the anti-correlation between the task-relevant feature dimension and the irrelevant feature dimensions, in high-information learning-blocks, systematically referring to an irrelevant feature dimension would yield an A-prime = 0 (see Appendix B, in the online supplemental, for data exclusion criteria).

## RESULTS

### OVERVIEW

There are two main objectives for the analysis: (i) Showing that the negative impact of ambiguous feedback on VCL is context dependent, most likely to be manifested in low-saliency conditions; (ii) Showing that participants’ performances in both low-saliency VCL tasks (mid-information and high-information) depended more on perceptual learning than their performances in the corresponding high-saliency VCL tasks.

### PRE-LEARNING PERFORMANCE

**FIGURE 5:**
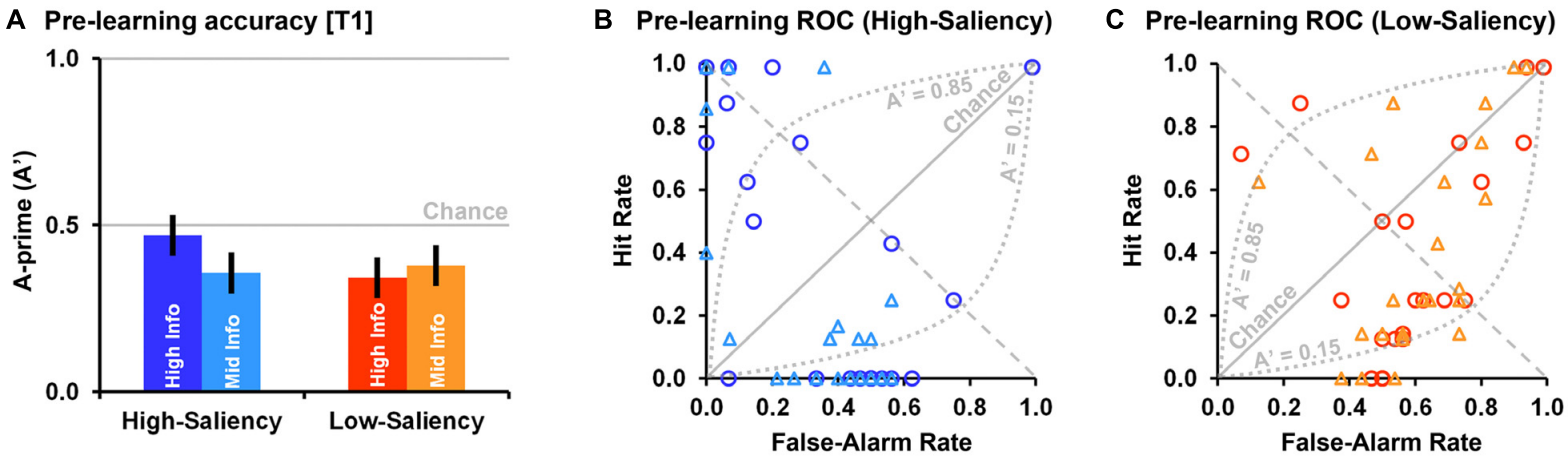
**(A)** Mean (error bars are SE of the mean) pre-learning A-prime scores in the high-information and mid-information feedback conditions (high-saliency vs. low-saliency). **(B)** Receiver Operation Characteristics (ROC) diagram for the high-saliency conditions (blue-purple circles = high-information; light blue triangles = mid-information). Each circle/triangle represents a single case. **(C)** ROC diagram for the low-saliency conditions (red circles = high-information; orange triangles = mid-information).

A two-way ANOVA with feature saliency and feedback information as independent variables, and categorization accuracy (A-prime) in the pre-learning test block (T1) as the dependent variable shows no significant interaction between feature saliency and feedback information prior to learning *F*(1,92) = 1.48, *p* = 0.23, no significant feedback information main effect *F*(1,92) = 0.39, and no feature saliency main effect *F*(1,92) = 0.74. This analysis confirms that the initial mean categorization accuracy in the four experimental conditions was the same (**Figure 5A**). A two-way ANOVA with feature saliency and feedback information as independent variables, and Hit rate in the pre-learning test block (T1) as the dependent variable shows, as well, no significant interaction between feature saliency and feedback information, *F*(1,92) = 2.35, *p* = 0.13, no significant feedback information main effect, *F*(1,92) = 1.29, *p* = 0.26, and no feature saliency main effect *F*(1,92) = 1.52, *p* = 0.22.

Differences between experimental conditions in pre-learning categorization patterns were evident in False-Alarm rates (the participants’ tendency to incorrectly identify two creatures as belonging to the same category). A two-way ANOVA with feature saliency and feedback information as independent variables, and participants’ False-Alarm rate in the pre-learning test block (T1) as the dependent variable, shows no significant interaction between feature saliency and feedback information prior to learning *F*(1,92) = 0.35, no significant feedback information main effect *F*(1,92) = 0.19, but a significant feature saliency main effect *F*(1,92) = 28.40, *p* < 0.00001, $\eta_{p}^{2}$ = 0.24. Specifically, the initial False-Alarm rate was greater in the low-saliency conditions (Mean = 0.61 ± SD = 0.20), where between stimuli differences were harder to detect, than in the high-saliency conditions (0.36 ± 0.24). This difference in False-Alarm rate was associated with participants’ response bias but had little impact on their overall categorization accuracy (A-prime score) due to corresponding differences in Hit rate (though these were statistically insignificant).

In the high-saliency conditions we find that most participants either exhibited high Hit rate and low False-Alarm rate indicating that they guessed the right feature dimension prior to the first learning-block, or near zero Hit rate and near 0.5 False-Alarm rate, which indicates participants that persistently categorized stimuli based on one of the two irrelevant feature dimensions. This suggests that in high-saliency conditions participants became quickly aware to the difference between stimuli. This is presented in **Figure 5B** as the area not confounded by the *0.85 > A-prime > 0.15* arches on the Receiver Operation Characteristics (ROC) diagram. A-prime scores higher than 0.85 or lower than 0.15 roughly match higher than 80% or lower than 20% correct, respectively (80% correct indicates cases with <6 errors in the first test-block). These indicate cases where a participant rapidly detected differences in one of the three feature dimensions in which stimuli varied. In the low-saliency conditions, on the other hand, many participants had both high Hit rate and high False-Alarm rate, where the vast majority of participants had closer to chance level performance (**Figure 5C**). That is, in the pre-learning phase, in low-saliency conditions, participants were much more likely to perceive paired creatures as belonging to the same-category, presumably due to not yet detecting the low-saliency differences in either the relevant or irrelevant feature dimensions.

**Table 1** shows the number of participants who exhibited high persistency in categorizing stimuli based on a specific feature dimension in the pre-learning phase, in each condition. A Fisher exact test shows that the portion of cases exhibiting high persistency in categorizing stimuli based on a specific feature dimension in the high-saliency conditions (28/48) was significantly higher (more than three times higher) than in the low-saliency conditions (9/48), *p* = 0.0001 (two-tailed). There were no significant differences in performance pattern between the mid-information and high-information conditions in either feature saliency level, both *p* > 0.4 (two-tailed, Fisher exact).

**Table 1:** Number of participants who showed accuracy levels of A-prime > 0.85 or A-prime < 0.15 in the pre-learning stage (24 participants in each condition).

Condition		A′ > 0.85	A′ < 0.15	High persistency
High-saliency	High-info	6/24	9/24	
	Mid-info	5/24	8/24	
	Total	11/48	17/48	28/48
Low-saliency	High-info	2/24	4/24	
	Mid-info	0/24	3/24	
	Total	2/48	7/48	9/48

*These A-prime values indicate participants that were highly persistent in categorizing creatures based on the predetermined feature dimension or one of the two irrelevant feature dimensions, respectively. An A-prime score of 0.85 roughly matches *d*-prime (*d*′) = 1.5, and is at about 80% correct.*

### INTERACTIVE IMPACT OF FEEDBACK INFORMATION AND FEATURE SALIENCY ON VCL DYNAMICS

We first compared the learning trajectories from the four conditions by accounting to the mean performances (A-prime scores) in the test and learning blocks combined (T1, L1, T2, L2, T3, L3, T4). A three-way ANOVA with feature saliency (low-saliency vs. high-saliency) and feedback information (mid-information vs. high-information) as independent between-subjects variables, test/learning-phase (T1, L1, T2, L2, T3, L3, T4) as a repeated measure variable, and performance (A-prime) as the dependent variable, shows a three-way interaction evident as a significant feature saliency by feedback information by learning-phase linear contrast (contrasting between the learning trajectories of the four conditions, assuming a simple linear model), *F*(1,92) = 3.77, *p* = 0.055, $\eta_{p}^{2}$ = 0.04. Additionally, this ANOVA shows a significant main effect of feature-saliency, *F*(1,92) = 10.23, *p* < 0.002, $\eta_{p}^{2}$ = 0.10 (high-saliency > low-saliency), and a trend toward a significant main effect of feedback information, *F*(1,92) = 3.67, *p* = 0.059, $\eta_{p}^{2}$ = 0.04 (high-information > mid-information). This ANOVA also shows that differences between the learning trajectories of the two feature saliency conditions are best explained by a cubic contrast, *F*(1,92) = 10.42, *p* < 0.002, $\eta_{p}^{2}$ = 0.10, indicating that differences between saliency conditions were maximized in the second test block (T2; unlike a linear contrast which indicates a monotonic change in differences between conditions as the VCL task progresses). There were no significant polynomial contrasts between the learning trajectories of the mid-information and high-information feedback conditions, all *p* > 0.15.

**FIGURE 6:**
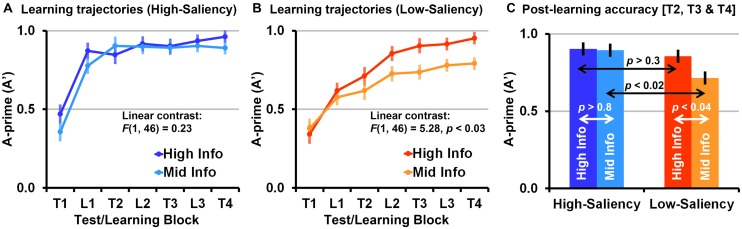
**(A)** Mean (error bars are SE of the mean) A-prime scores in all blocks (T1, L1, T2, L2, T3, L3, T4) in the high-saliency conditions. **(B)** Mean A-prime scores in all blocks in the low-saliency conditions. **(C)** Mean post-learning A-prime scores (mean of T2, T3, and T4) in the high-information and mid-information feedback conditions (high-saliency vs. low-saliency).

*Post hoc* analyses show that the above three-way interaction primarily results from slower learning and overall lower categorization performances in the low-saliency mid-information condition. A two-way ANOVA comparing the two low-saliency conditions, with feedback information as an independent variables, learning-phase (T1, L1, T2, L2, T3, L3, T4) as a repeated measure variable, and performance (A-prime) as the dependent variable, shows a significant linear contrast between the mid- and high-information feedback conditions, *F*(1,46) = 5.28, *p* < 0.03, $\eta_{p}^{2}$ = 0.10, a trend towards a significant feedback information simple main effect when accounting to all blocks, *F*(1,46) = 3.41, *p* = 0.071, $\eta_{p}^{2}$ = 0.07, and a significant feedback information simple main effect when accounting only to the blocks starting from T2 (following the first learning block), *F*(1,46) = 4.84, *p* < 0.04, $\eta_{p}^{2}$ = 0.09. This indicates faster learning (steeper ascending slope) and overall better performance in the low-saliency high-information condition than in the low-saliency mid-information condition (**Figure 6B**). A two-way ANOVA compering the two mid-information conditions shows that mean performance in the low-saliency mid-information condition was lower than in the high-saliency mid-information condition. This was evident as a significant quadratic contrast, *F*(1,46) = 8.15, *p* < 0.007, $\eta_{p}^{2}$ = 0.15, and a significant feature saliency simple main effect, *F*(1,46) = 6.35, *p* < 0.02, $\eta_{p}^{2}$ = 0.12.

**FIGURE 7:**
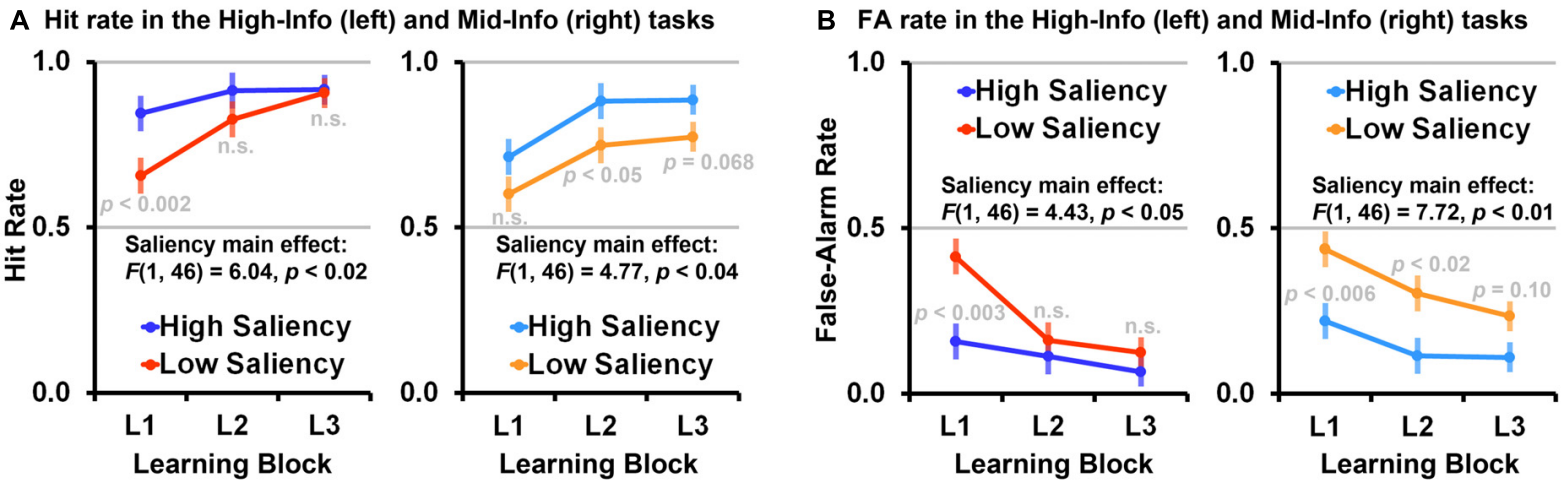
**(A)** Differences in mean (error bars are SE of the mean) Hit rates in the learning blocks (L1, L2, L3) attributed to the saliency manipulation, in the high-information (left) and mid-information (right) conditions. **(B)** Differences in mean False-Alarm rates (n.s. indicates *p* > 0.10).

In the high-saliency conditions there was no difference between the mid-information and high-information feedback conditions, *F*(1,46) = 0.67, indicating that learning in high-saliency conditions was not significantly affected by the feedback ambiguity manipulation (**Figure 6A**). Importantly, we found that learning in the high-saliency high-information condition was better than in the low-saliency high-information condition. This was evident as a significant linear contrast, *F*(1,46) = 6.34, *p* < 0.02, $\eta_{p}^{2}$ = 0.12, and a significant feature saliency simple main effect, *F*(1,46) = 3.88, *p* = 0.055, $\eta_{p}^{2}$ = 0.08, indicating faster learning and overall better performance in the high-information high-saliency condition than in the high-information low-saliency condition. That is, perceptual learning played a significant role in both low-saliency conditions. Nevertheless, unlike the low-saliency mid-information condition where learning was fairly impaired throughout the entire task, in the low-saliency high-information condition later-phase performances matched the performances observed in the two high-saliency conditions (see also **Figure 7**).

The following analysis is based only on the mean performances in the four test blocks, where the composition of trials in the two feedback information conditions (for each given feature saliency level) was identical. This analysis shows that the exceptionally lower performance in the low-saliency mid-information VCL condition was evident as lower mean performance in all post-learning test trials (T2, T3, T4). A three-way ANOVA with feature saliency (low-saliency vs. high-saliency) and feedback information (mid-information vs. high-information) as independent variables, learning-phase (pre-learning [T1] versus post-learning [T2, T3, T4]) as a repeated measure variable, and categorization accuracy (A-prime) as the dependent variable, shows a significant three-way interaction between feature-saliency, feedback information, and learning-phase, *F*(1,92) = 4.29, *p* < 0.05, $\eta_{p}^{2}$ = 0.045 (Greenhouse–Geisser corrected).

*Post hoc* analyses show that the post-learning mean performance in the low-saliency mid-information condition was lower than all other conditions, whereas the mean performance in the three other conditions was largely matched (**Figure 6C**). When contrasting between the two high-saliency conditions we found no simple main effect of feedback information, *F*(1,46) = 0.02, but we did find a simple main effect of feedback information when contrasting the two low-saliency conditions, *F*(1,46) = 4.84, *p* < 0.04, $\eta_{p}^{2}$ = 0.09. When contrasting between the two high-information conditions we found no significant simple main effects of feature saliency, *F*(1,46) = 0.95, but we found a simple main effects of feature saliency when contrasting the two mid-information conditions *F*(1,46) = 6.79, *p* < 0.02, $\eta_{p}^{2}$ = 0.13. Post-learning performances in all four conditions were above chance (A-prime = 0.5), all *p* < 0.005. There were no significant pre-learning (T1) simple main effects, all *p* > 0.15 (**Figure 5A**).

### PERCEPTUAL LEARNING

To further investigate the role of perceptual learning in low-saliency VCL, we looked at the impact of the feature saliency manipulation on the participants’ Hit rate and False-Alarm rate in the learning blocks of each feedback information condition, taking advantage of the composition of these trials. Specifically, in high-information learning trials the task-relevant feature dimension was the only feature dimension discriminating between different-categories paired creatures, and thus performance in these trials is less affected by having several features with potential discriminative value competing for attention.

A three-way ANOVA with feature saliency (low-saliency vs. high-saliency) and feedback information (mid-information vs. high-information) as independent between-subjects variables, learning-phase (L1, L2, L3) as a repeated measure variable, and Hit rate as the dependent variable, shows a trend toward a significant feature saliency by feedback information by learning-phase linear contrast, *F*(1,92) = 3.19, *p* = 0.077, $\eta_{p}^{2}$ = 0.03, a significant main effect of feature saliency, *F*(1,92) = 10.26, *p* < 0.002, $\eta_{p}^{2}$ = 0.10 (high-saliency > low-saliency), and a significant main effect of feedback information, *F*(1,92) = 5.26, *p* < 0.03, $\eta_{p}^{2}$ = 0.05 (high-information > mid-information). This ANOVA also shows differences between the learning trajectories of the two feature saliency conditions evident as a trend toward a significant linear contrast, *F*(1,92) = 3.36, *p* = 0.07, $\eta_{p}^{2}$ = 0.03. There were no significant polynomial contrasts between the learning trajectories of the mid-information and high-information feedback conditions, all *p* > 0.35. Largely, this ANOVA mirrors the effects observed in the above analysis with A-prime as the dependent variable (**Figure 7A**).

A three-way ANOVA with feature saliency (low-saliency vs. high-saliency) and feedback information (mid-information vs. high-information) as independent between-subjects variables, learning-phase (L1, L2, L3) as a repeated measure variable, and False-Alarm rate as the dependent variable, shows a trend toward a significant feature saliency by feedback information by learning-phase linear contrast, *F*(1,92) = 3.19, *p* = 0.098, $\eta_{p}^{2}$ = 0.03, and a main effect of feature saliency, *F*(1,92) = 12.26, *p* < 0.001, $\eta_{p}^{2}$ = 0.12 (low-saliency > high-saliency). There was no significant feedback information main effect, *F*(1,92) = 2.20, *p* = 0.14. This ANOVA also shows a feature saliency main effect evident as a significant linear contrast, *F*(1,92) = 7.59, *p* < 0.007, $\eta_{p}^{2}$ = 0.08 (with a steeper descending slope in the low-saliency tasks due to low False-Alarm rate in the high-saliency tasks, starting at L1; a close to floor effect). There were no significant polynomial contrasts between the learning trajectories of the mid-information and high-information feedback conditions, all *p* > 0.50 (**Figure 7B**).

Unlike the Hit rate, which was significantly affected both by the feature saliency and feedback information manipulations, the False-Alarm rate was primarily affected by the feature saliency manipulation with substantially higher False-Alarm rate in low saliency tasks. This indicates, as might be expected, that low-saliency primarily (yet not exclusively) compromise VCL by reducing the capacity to differentiate between stimuli from two distinct categories.

### CATEGORIZATION PERFORMANCE IN THE CONTROL TASKS

A three-way ANOVA with feature saliency (low-saliency vs. high-saliency) and feedback-availability (no-information feedback vs. unsupervised categorization with no feedback) as independent variables, learning-phase [pre-learning (T1) vs. post-learning (T2, T3, T4)] as a repeated measure variable, and categorization accuracy (A-prime) as the dependent variable, shows no learning main effect (pre-learning to post-learning), *F*(1,93) = 0.13, *p* > 0.7, or any other significant main effect or interaction effect, all *p* > 0.1 (see **Table 2**). This analysis confirms that the categorization accuracies and the performance improvement observed in the four experimental conditions (mid-information and high-information) could only be attributed to feedback information, and not to cumulative experience with the stimuli. We note that in the control tasks participants had greater tendency not to respond on time, or to exhibit stereotypical persistent response pattern (“yea-sayers” or “nay-sayers”). Being relatively common, cases with such performance patterns were not excluded from this analysis.

**Table 2:** Mean (±SD) A-prime in the control tasks with no-information feedback and unsupervised categorization tasks where no feedback was provided.

Condition		Pre [T1]	Post [T2, T3, and T4]
High-saliency	No-info	0.31 ± 0.30	0.31 ± 0.31
	Unsupervised	0.38 ± 0.35	0.45 ± 0.41
Low-saliency	No-info	0.40 ± 0.30	0.39 ± 0.37
	Unsupervised	0.27 ± 0.15	0.23 ± 0.23

*Here we find no pre-learning to post-learning performance changes.*

## DISCUSSION

We tested the interaction between feature saliency and feedback information in visual category learning (VCL) tasks as a mean to explore interactions between perceptual learning and attentional learning processes in different VCL scenarios (high-saliency vs. low-saliency scenarios). We found that in high-saliency tasks participants reached highest accuracies within the first learning-block, surprisingly also in the mid-information feedback VCL task where learning trial were ambiguous (**Figure 6A**). In the low-saliency high-information feedback condition we found that VCL required more learning trials than in the high-saliency high-information condition. Nevertheless, when having high-information feedback available, participants ultimately reached the same categorization accuracies observed in the two high-saliency tasks (**Figures 6B and 7A,B-left**).

Importantly, as we hypothesized, we found that ambiguous feedback substantially impaired VCL in low-saliency tasks, resulting in significantly lower categorization accuracies than those observed in the three other experimental conditions (**Figure 6C**). We suggest that in low-feature-saliency scenarios with ambiguous feedback, learning is more difficult due to a “chicken-or-the-egg” cognitive loop paradox. In such VCL scenarios there are two cognitive challenges that need to be resolved: (i) becoming aware to important feature-wise differences between stimuli and increasing sensitivity to these differences, (ii) and determining which feature-dimensions are relevant for categorization. The paradox is that resolving each challenge depends on resolving the other challenge first. On the one hand, to be effective, perceptual learning requires persistently directing attention to specific feature dimension (Goldstone, 1994; Gilbert et al., 2001; Kruschke, 2001); but this is unlikely to happen when there is an ambiguity regarding which feature dimension needs to be attended. On the other hand, attentional learning requires using feedback information for systematically valuing which feature dimension is important for categorization, while ignoring irrelevant feature dimensions; but attentional learning is likely to be compromised when there are only vague impressions of any differences between the perceived objects.

Challenges distinct to low-saliency VCL scenarios were already evident as differences in performance patterns between the high-saliency and low-saliency conditions prior to learning (T1): In high-saliency conditions, most participants showed high persistency in categorizing the creatures based on one of the three feature dimensions in which the creatures varied (**Table 1**; **Figure 5B**). The frequently observed *A-prime > 0.85* or *A-prime < 0.15* scores indicates that in high-saliency tasks, prior to learning, most participants were likely to instantly detect at least one of the three differentiating feature dimensions and to consistently use it for categorizing the creatures. We can assume that in the pre-learning phase, in high-saliency tasks, participants asked themselves “which of these differentiating attributes is most relevant for categorizing these creatures?” On the other hand, in low-saliency conditions most participants performed closer to chance level (A-prime = 0.5), where many participants had both high Hit rate and high False-Alarm rate. This indicates that in low-saliency tasks participants perceived most paired creatures as being the same, presumably not detecting any differences (**Table 1**; **Figure 5C**). Here many participants may have asked themselves “do these creatures differ at all, and if yes, in what way?” These two distinct starting points impose different constraints on the cognitive strategies that participants could employ during later VCL phases.

In high-saliency tasks, in the first learning block (L1), we found that participants could use the feedback information from few trials for quickly inferring if the feature dimension by which they categorized the creatures prior to learning (T1) is the relevant feature dimension. This was true even when provided with mid-information feedback. If the feedback indicated frequent errors, attention could be quickly redirected to one of the other two salient feature dimensions by which creatures varied. In high-saliency tasks there was little or no need for perceptual learning to take place, and the primary cognitive challenge in these tasks was testing hypotheses whether one feature or the other is relevant for categorization. This enabled the rapid inference of a generalized categorization rule.

In low-saliency VCL tasks the detection of feature dimensions in which the creatures varied was initially much harder than in high-saliency tasks. Thus, it was most unlikely that during the pre-learning test block (T1) the participants would form a solid ‘hypothesis space’ where there are few alternative categorization rules to choose from. In the first learning block (L1), instead of using the feedback for systematically testing which feature dimension is relevant for categorization, participants primarily used the feedback for perceptual learning and for further detection of possibly other diagnostic feature dimensions. The feedback enabled cumulating evidences indicating that apparently identical paired creatures are in fact different. This forced participants to continue searching for diagnostic feature dimensions, as it also enabled them to validate if some vague *perceived* differences between creatures represent concrete differences in a specific feature dimension that deserve being further attended. This enabled increasing the sensitivity to this feature dimension and to reduce categorization error rate (Herzog and Fahle, 1997).

Being provided with mid-information feedback increased the odds that few vague impressions of possibly differentiating feature dimensions would be simultaneously considered as relevant for categorizing creatures. Not attending to only one low-saliency feature dimension at a time was likely to compromise perceptual learning, and thus it hindered the increase in sensitivity to partially attended feature dimensions. In turn, not becoming sufficiently sensitive to differences in a given feature dimension reduced the participants’ confidence that this feature dimension has diagnostic value, further reducing the odds that it would be frequently attended, and thus further compromising perceptual learning.

It is also possible that in low-saliency tasks some participants attended a specific feature dimension of the creatures (e.g., the creatures heads) across multiple successive trials, even prior to learning (T1). If this feature was one of the three feature dimensions in which stimuli varied, this could result in the participant becoming more sensitive to between-creatures differences in this feature dimension even without receiving feedback (Petrov et al., 2006; Liu et al., 2012). If the feature dimension relevant for categorization was consistently attended during T1, such accidental direction of attention could enable faster learning. However, if one of the two irrelevant feature dimensions was consistently attended during T1, increased sensitivity to this feature dimension could have stunted the learning of the task-relevant feature dimension, resulting in slower VCL (Kruschke, 2003; Kruschke et al., 2005; Bott et al., 2007; Blair et al., 2009).

In low-saliency tasks, providing participants with high-information feedback in L1 could allow participants an opportunity to confirm if the initially attended (yet still poorly represented) feature dimension is the one relevant for categorization. However, due to relatively poor representation, this learning was not as fast as learning in high-saliency conditions since it required either slow increase in sensitivity to the earlier detected differences, or the search for other low-saliency differences between creatures. This should have been followed by further perceptual learning for increasing sensitivity to the detected differences. If the feature dimension most attended in T1 was one of the two irrelevant feature dimensions (the more likely scenario), the introduction of mid-information feedback in L1 was likely to have an initial negative impact on VCL, reinforcing the impression that an irrelevant low-saliency feature dimension is in fact relevant for categorization. This could prevent the participant from searching for the task relevant feature dimension.

Despite the suggested cognitive loop paradox, we found that learning in the low-saliency mid-information condition was ultimately feasible, with above chance mean performance (**Figure 6C**). This can be explained, at least partially, by characteristics of the current experimental design. First, low-saliency was not ‘too low’ – based on the pre-learning (T1) performances in the low-saliency tasks, it is clear that differences between paired creatures were hard to detect (harder than in the high-saliency tasks) within the short stimuli presentation time. Nevertheless, in the low-saliency, high-information condition we found that participants eventually reached performance levels matching those observed in the high-saliency conditions (though it required more learning trials, **Figures 6 and 7**). This suggests that the current experimental design does not fully exhaust perceptual learning capacities of normal adults.

Secondly, in the current experimental design ambiguous feedback was not ‘too ambiguous’ – based on performances in the high-saliency mid-information condition, it is clear that when having a total of three feature dimensions in which stimuli vary, and when in each given trial the task-relevant feature dimension is competing for the participant’s attention with only one similarly salient irrelevant feature dimension, feedback ambiguity is manageable and in fact seem to have negligible evident impact on VCL performances. This is clearly not expected to be the case in VCL tasks with even more ambiguous feedback. Future studies may investigate the interaction between feature saliency and feedback information in more ‘extreme’ feedback ambiguity conditions. For example, if increasing the total number of varying feature dimensions to five, where in each trial the paired creatures differ in the task-relevant feature dimension and a random combination of three of the irrelevant feature dimensions, learning the categorization rule may (would) take longer even in high-saliency conditions. Moreover, in such scenarios it may become more likely that learning in low-saliency VCL tasks would be utterly compromised due to having attention being directed to the task relevant feature dimension even less frequently than in the mid-information conditions that we tested here. In such scenarios it would become even less likely that brain activation associated with task-relevant features would reach the threshold required for significant changes in representation to take place (Goldstone, 1994; Gilbert et al., 2001; Kruschke, 2001).

The reported differences in performances between the low-saliency mid-information condition and the low-saliency high-information condition may contribute to the ongoing debate regarding the role of attention control in perceptual learning, to which we referred in more detail in the Introduction (Ahissar and Hochstein, 1993; Schoups et al., 2001; Herzog and Fahle, 2002; Seitz et al., 2009; Kourtzi, 2010; Roelfsema et al., 2010; Aberg and Herzog, 2012). Here we show that VCL performance in the low-saliency mid-information condition, where perceptual learning had to take place under conditions that required distributing attention among few feature dimensions, was most impaired. Nevertheless our data does not allow directly testing the degree to which participants’ sensitivity to the relevant and irrelevant feature dimensions has been changed following learning, apart from how these changes impacted categorization performances (which are likely to reflect changes in representation of the task-relevant feature dimension). Testing participants’ sensitivity to differences between paired creatures in each feature dimension separately, before and after VCL has been completed, may indicate if perceptual learning took place, if it is restricted to the task-relevant feature dimension, or if it is evident as increased sensitivity also to task-irrelevant feature dimensions (we advise having a short pre-learning sensitivity test, since extensive exposure to the stimuli may facilitate unsupervised perceptual learning to some arbitrary features in which stimuli vary).

Related to the above, we suggest that as much as ambiguous feedback may compromise VCL, it is more likely to result in an increased perceptual sensitivity to irrelevant feature dimensions. This is due to greater correlations between the task-relevant feature dimension and the irrelevant ones, which is likely to result in more frequent associations between irrelevant feature dimensions and the feedback (or reward) that follows a categorization decision. This may reinforce irrelevant feature dimensions more frequently than in higher-information feedback conditions, where only the task-relevant feature dimension is frequently reinforced. For example, when ‘*A*’ denotes the relevant feature-dimension, ‘*B,*’ *‘C,’ ‘D,’* and ‘*E*’ the irrelevant feature-dimensions and ‘*X*’ the categorization decision outcome, in a scenario with trials composition such as (A∪B → X) ∩ (A∪D → X) ∩ (A∪C → X) ∩ (A∪E → X) ∩ (A∪D → X)⋯ there would be relatively sparse association between each of the irrelevant feature-dimensions and the decision outcome (25% of the trials; -log _2_2/5 = 1.322 bits of information per trial). On the other hand, in a scenario with trial composition such as (A∪B∪C∪D → X)∩(A∪C∪D∪E → X)∩(A∪B∪C∪D → X)∩(A∪B∪C∪E → X)∩(A∪B∪D∪E → X)⋯ (75% of the trials; -log _2_4/5 = 0.322 bits per trial) it would become more likely that at least some of the task-irrelevant feature-dimensions would be reinforced following VCL.

That is, the administration of learning trials and their informativeness may greatly impact the effectiveness of VCL and, the odds that VCL would result in evident changes in neural representation of task-irrelevant feature dimensions (see Jiang et al., 2007; Folstein et al., 2013, 2014, for related findings). More generally, we predict that in order to observe VCL associated with an increased sensitivity to irrelevant feature dimensions, the VCL task has to meet two requirements: (i) the number of irrelevant feature dimensions should be small, and (ii) assuming a given small number of irrelevant feature dimensions, the feedback information in VCL tasks should be minimized so to increase the odds that attention would be distributed between these few irrelevant feature dimensions, so that at least some of these feature dimensions would be frequently reinforced (see Watanabe and Sasaki, 2015, for a related discussion).

Finally, future studies may also investigate how VCL, under different feature saliency conditions, can be optimized while accounting for the fact that when categorizing same-category stimuli, the information gained is not with the same use as the information gained when categorizing different-category stimuli (even when receiving the same information quantity). Specifically, informative same-category stimuli comparison is most effective for highlighting the permitted within category variability, whereas informative different-categories stimuli comparison highlights between categories differences (Hammer et al., 2009a,b, 2010; Kurtz et al., 2013; Carvalho and Goldstone, 2014). For this reason, in high-saliency VCL tasks comparison of same-category examples would be valuable for learning which feature dimensions should be ignored (contributing to attentional learning). On the other hand, comparison of same-category examples would not have much value in low-saliency VCL tasks where the main challenge is to detect important subtle differences between stimuli and to become more sensitive to these differences (requiring perceptual learning). In such scenarios, informative comparison of different-categories exemplars would be most critical to VCL. These ideas have implications for understanding possible differences between general neurocognitive mechanisms of VCL, versus those that are specifically critical for acquiring expertise (Scott et al., 2008).

In sum, the current study, despite its limitations resulting from not exploring a broader range of feature saliency and feedback ambiguity, provides a methodological and theoretical framework that if adopted may contribute to the understanding of how neurocognitive mechanisms of perception, attention, reasoning and learning are jointly being used, or failed to be used, in varying VCL scenarios.

## Conflict of Interest Statement

The authors declare that the research was conducted in the absence of any commercial or financial relationships that could be construed as a potential conflict of interest.

## References

[B1] Aberg K. C. Herzog M. H. (2012). Different types of feedback change decision criterion and sensitivity differently in perceptual learning. *J. Vis.* 12 3 10.1167/12.3.3 22396463

[B2] Ahissar M. Hochstein S. (1993). Attentional control of early perceptual learning. *Proc. Natl. Acad. Sci. U.S.A.* 90 5718–5722 10.1073/pnas.90.12.5718 8516322 PMC46793

[B3] Ahissar M. Hochstein S. (1997). Task difficulty and the specificity of perceptual learning. *Nature* 387 401–406 10.1038/387401a0 9163425

[B4] Antzoulatos E. G. Miller E. K. (2011). Differences between neural activity in prefrontal cortex and striatum during learning of novel abstract categories. *Neuron* 71 243–249 10.1016/j.neuron.2011.05.040 21791284 PMC3253019

[B5] Awh E. Belopolsky A. V. Theeuwes J. (2012). Top-down versus bottom-up attentional control: a failed theoretical dichotomy. *Trends Cogn. Sci.* 16 437–443 10.1016/j.tics.2012.06.010 22795563 PMC3426354

[B6] Awh E. Jonides J. (2001). Overlapping mechanisms of attention and spatial working memory. *Trends Cogn. Sci.* 5 119–126 10.1016/S1364-6613(00)01593-X 11239812

[B7] Baluch F. Itti L. (2011). Mechanisms of top-down attention. *Trends Neurosci.* 34 210–224 10.1016/j.tins.2011.02.003 21439656

[B8] Blair M. R. Watson M. R. Walshe R. C. Maj F. (2009). Extremely selective attention: eye-tracking studies of the dynamic allocation of attention to stimulus features in categorization. *J. Exp. Psychol. Learn. Mem. Cogn.* 35 1196 10.1037/a0016272 19686015

[B9] Borji A. Itti L. (2013). State-of-the-art in visual attention modeling. *IEEE Trans. Pattern Anal. Mach. Intell.* 35 185–207 10.1109/TPAMI.2012.89 22487985

[B10] Bott L. Hoffman A. B. Murphy G. L. (2007). Blocking in category learning. *J. Exp. Psychol. Gen.* 136 685 10.1037/0096-3445.136.4.685 PMC2323587 17999579

[B11] Carvalho P. F. Goldstone R. L. (2014). Effects of interleaved and blocked study on delayed test of category learning generalization. *Front. Psychol.* 5:936 10.3389/fpsyg.2014.00936 PMC4141442 25202296

[B12] Chen L. Meier K. M. Blair M. R. Watson M. R. Wood M. J. (2013). Temporal characteristics of overt attentional behavior during category learning. *Atten. Percept. Psychophys.* 75 244–256 10.3758/s13414-012-0395-8 23151960

[B13] Chin-Parker S. Ross B. H. (2004). Diagnosticity and prototypicality in category learning: a comparison of inference learning and classification learning. *J. Exp. Psychol. Learn. Mem. Cogn.* 30 216 10.1037/0278-7393.30.1.216 14736308

[B14] Corbetta M. Patel G. Shulman G. L. (2008). The reorienting system of the human brain: from environment to theory of mind. *Neuron* 58 306–324 10.1016/j.neuron.2008.04.017 18466742 PMC2441869

[B15] Daniel R. Pollmann S. (2010). Comparing the neural basis of monetary reward and cognitive feedback during information-integration category learning. *J. Neurosci.* 30 47–55 10.1523/JNEUROSCI.2205-09.2010 20053886 PMC6632509

[B16] Davis T. Love B. C. Preston A. R. (2012). Striatal and hippocampal entropy and recognition signals in category learning: simultaneous processes revealed by model-based fMRI. *J. Exp. Psychol. Learn. Mem. Cogn.* 38 821 10.1037/a0027865 PMC3403290 22746951

[B17] Diesendruck G. Hammer R. Catz O. (2003). Mapping the similarity space of children and adults’ artifact categories. *Cogn. Dev.* 18 217–231 10.1016/S0885-2014(03)00021-2

[B18] Fahle M. Poggio T. (eds) (2002). *Perceptual Learning.* Cambridge, MA: MIT Press.

[B19] Folstein J. R. Palmeri T. J. Gauthier I. (2013). Category learning increases discriminability of relevant object dimensions in visual cortex. *Cereb. Cortex* 23 814–823 10.1093/cercor/bhs067 22490547 PMC3593573

[B20] Folstein J. R. Palmeri T. J. Gauthier I. (2014). Perceptual advantage for category-relevant perceptual dimensions: the case of shape and motion. *Front. Psychol.* 5:1394 10.3389/fpsyg.2014.01394 PMC4249057 25520691

[B21] Gazzaley A. Nobre A. C. (2012). Top-down modulation: bridging selective attention and working memory. *Trends Cogn. Sci.* 16 129–135 10.1016/j.tics.2011.11.014 22209601 PMC3510782

[B22] Gilbert C. D. Sigman M. Crist R. E. (2001). The neural basis of perceptual learning. *Neuron* 31 681–697 10.1016/S0896-6273(01)00424-X 11567610

[B23] Goldstone R. L. (1994). Influences of categorization on perceptual discrimination. *J. Exp. Psychol. Gen.* 123 178–200 10.1037/0096-3445.123.2.178 8014612

[B24] Goldstone R. L. (1998). Perceptual learning. *Annu. Rev. Psychol.* 49 585–612 10.1146/annurev.psych.49.1.585 9496632

[B25] Goldstone R. L. Lippa Y. Shiffrin R. M. (2001). Altering object representations through category learning. *Cognition* 78 27–43 10.1016/S0010-0277(00)00099-8 11062321

[B26] Goldstone R. L. Steyvers M. (2001). The sensitization and differentiation of dimensions during category learning. *J. Exp. Psychol. Gen.* 130 116 10.1037/0096-3445.130.1.116 11293456

[B27] Greenhouse S. W. Geisser S. (1959). On methods in the analysis of profile data. *Psychometrika* 24 95–112 10.1007/BF02289823

[B28] Grier J. B. (1971). Nonparametric indexes for sensitivity and bias: computing formulas. *Psychol. Bull.* 75 424 10.1037/h0031246 5580548

[B29] Hammer R. Bar-Hillel A. Hertz T. Weinshall D. Hochstein S. (2008). Comparison processes in category learning: from theory to behavior. *Brain Res.* 1225 102–118 10.1016/j.brainres.2008.04.079 18614160

[B30] Hammer R. Brechmann A. Ohl F. Weinshall D. Hochstein S. (2010). Differential category learning processes: the neural basis of comparison-based learning and induction. *Neuroimage* 52 699–709 10.1016/j.neuroimage.2010.03.080 20363336

[B31] Hammer R. Diesendruck G. (2005). The role of dimensional distinctiveness in children’s and adults’ artifact categorization. *Psychol. Sci.* 16 137–144 10.1111/j.0956-7976.2005.00794.x 15686580

[B32] Hammer R. Diesendruck G. Weinshall D. Hochstein S. (2009a). The development of category learning strategies: what makes the difference? *Cognition* 112 105–119 10.1016/j.cognition.2009.03.012 19426967

[B33] Hammer R. Hertz T. Hochstein S. Weinshall D. (2009b). Category learning from equivalence constraints. *Cogn. Process.* 10 211–232 10.1007/s10339-008-0243-x 19050949

[B34] Hammer R. Hertz T. Hochstein S. Weinshall D. (2007). “Classification with positive and negative equivalence constraints: theory, computation and human experiments,” in *Advances in Brain, Vision, and Artificial Intelligence, Lecture Notes in Computer Science,* eds Mele F. Ramella G. Santillo S. Ventriglia F. (Berlin Heidelberg: Springer-Verlag Press), 264–276.

[B35] Hammer R. Sloutsky V. Grill-Spector K. (2012). “The interplay between feature saliency and feedback information in visual category learning tasks,” *Proceedings to the 34th Annual Conference of the Cognitive Science Society*, Sapporo. PMC4208067 25346948

[B36] Herzog M. H. Fahle M. (1997). The role of feedback in learning a vernier discrimination task. *Vision Res.* 37 2133–2141 10.1016/S0042-6989(97)00043-6 9327060

[B37] Herzog M. H. Fahle M. (2002). Effects of grouping in contextual modulation. *Nature* 415 433–436 10.1038/415433a 11807555

[B38] Hoffman A. B. Rehder B. (2010). The costs of supervised classification: the effect of learning task on conceptual flexibility. *J. Exp. Psychol. Gen.* 139 319 10.1037/a0019042 20438254

[B39] Itti L. Koch C. Niebur E. (1998). A model of saliency-based visual attention for rapid scene analysis. *IEEE Trans. Pattern Anal. Mach. Intell.* 20 1254–1259 10.1109/34.730558

[B40] Jiang X. Bradley E. Rini R. A. Zeffiro T. VanMeter J. Riesenhuber M. (2007). Categorization training results in shape-and category-selective human neural plasticity. *Neuron* 53 891–903 10.1016/j.neuron.2007.02.015 17359923 PMC1989663

[B41] Koch C. Tsuchiya N. (2007). Attention and consciousness: two distinct brain processes. *Trends Cogn. Sci.* 11 16–22 10.1016/j.tics.2006.10.012 17129748

[B42] Kourtzi Z. (2010). Visual learning for perceptual and categorical decisions in the human brain. *Vision Res.* 50 433–440 10.1016/j.visres.2009.09.025 19818361

[B43] Kruschke J. K. (2001). Toward a unified model of attention in associative learning. *J. Math. Psychol.* 45 812–863 10.1006/jmps.2000.1354

[B44] Kruschke J. K. (2003). Attention in learning. *Curr. Dir. Psychol. Sci.* 12 171–175 10.1111/1467-8721.01254

[B45] Kruschke J. K. Blair N. J. (2000). Blocking and backward blocking involve learned inattention. *Psychon. Bull. Rev.* 7 636–645 10.3758/BF03213001 11206204

[B46] Kruschke J. K. Kappenman E. S. Hetrick W. P. (2005). Eye gaze and individual differences consistent with learned attention in associative blocking and highlighting. *J. Exp. Psychol. Learn. Mem. Cogn.* 31 830 10.1037/0278-7393.31.5.830 16248737

[B47] Kurtz K. J. Boukrina O. Gentner D. (2013). Comparison promotes learning and transfer of relational categories. *J. Exp. Psychol. Learn. Mem. Cogn.* 39 1303 10.1037/a0031847 23421515

[B48] LaBar K. S. Gitelman D. R. Parrish T. B. Mesulam M. (1999). Neuroanatomic overlap of working memory and spatial attention networks: a functional MRI comparison within subjects. *Neuroimage* 10 695–704 10.1006/nimg.1999.0503 10600415

[B49] Liu J. Lu Z. L. Dosher B. A. (2012). Mixed training at high and low accuracy levels leads to perceptual learning without feedback. *Vision Res.* 61 15–24 10.1016/j.visres.2011.12.002 22227159 PMC3330187

[B50] Lopez-Paniagua D. Seger C. A. (2011). Interactions within and between corticostriatal loops during component processes of category learning. *J. Cogn. Neurosci.* 23 3068–3083 10.1162/jocn_a_00008 21391766

[B51] Love B. C. (2002). Comparing supervised and unsupervised category learning. *Psychon. Bull. Rev.* 9 829–835 10.3758/BF03196342 12613690

[B52] Lupyan G. Rakison D. H. McClelland J. L. (2007). Language is not just for talking redundant labels facilitate learning of novel categories. *Psychol. Sci.* 18 1077–1083 10.1111/j.1467-9280.2007.02028.x 18031415

[B53] Maddox W. T. Ashby F. G. Bohil C. J. (2003). Delayed feedback effects on rule-based and information-integration category learning. *J. Exp. Psychol. Learn. Mem. Cogn.* 29 650 10.1037/0278-7393.29.4.650 12924865

[B54] Mathy F. Haladjian H. H. Laurent E. Goldstone R. L. (2013). Similarity-dissimilarity competition in disjunctive classification tasks. *Front. Psychol.* 4:26 10.3389/fpsyg.2013.00026 PMC3567436 23403979

[B55] McColeman C. M. Barnes J. I. Chen L. Meier K. M. Walshe R. C. Blair M. R. (2014). Learning-induced changes in attentional allocation during categorization: a sizable catalog of attention change as measured by eye movements. *PLoS ONE* 9:e83302 10.1371/journal.pone.0083302 PMC3908863 24497915

[B56] Namy L. L. Gentner D. (2002). Making a silk purse out of two sow’s ears: young children’s use of comparison in category learning. *J. Exp. Psychol. Gen.* 131 5–15 10.1037/0096-3445.131.1.5 11900103

[B57] Nosofsky R. M. (1984). Choice, similarity, and the context theory of classification. *J. Exp. Psychol. Learn. Mem. Cogn.* 10 104–114 10.1037/0278-7393.10.1.104 6242730

[B58] Nosofsky R. M. (1986). Attention, similarity, and the identification–categorization relationship. *J. Exp. Psychol. Gen.* 115 39–61 10.1037/0096-3445.115.1.39 2937873

[B59] Nosofsky R. M. Palmeri T. J. (1996). Learning to classify integral-dimension stimuli. *Psychon. Bull. Rev.* 3 222–226 10.3758/BF03212422 24213871

[B60] Petrov A. A. Dosher B. A. Lu Z. L. (2006). Perceptual learning without feedback in non-stationary contexts: data and model. *Vision Res.* 46 3177–3197 10.1016/j.visres.2006.03.022 16697434

[B61] Rehder B. Hoffman A. B. (2005). Thirty-something categorization results explained: selective attention, eye-tracking, and models of category learning. *J. Exp. Psychol. Learn. Mem. Cogn.* 31 811–829 10.1037/0278-7393.31.5.811 16248736

[B62] Roelfsema P. R. van Ooyen A. Watanabe T. (2010). Perceptual learning rules based on reinforcers and attention. *Trends Cogn. Sci.* 14 64–71 10.1016/j.tics.2009.11.005 20060771 PMC2835467

[B63] Rosch E. Mervis C. B. (1975). Family resemblances: studies in the internal structure of categories. *Cogn. Psychol.* 7 573–605 10.1016/0010-0285(75)90024-9

[B64] Schoups A. Vogels R. Qian N. Orban G. (2001). Practicing orientation identification improves orientation coding in V1 neurons. *Nature* 412 549–553 10.1038/35087601 11484056

[B65] Schulz L. E. Bonawitz E. B. Griffiths T. L. (2007). Can being scared cause tummy aches? Naive theories, ambiguous evidence, and preschoolers’ causal inferences. *Dev. Psychol.* 43 1124–1139 10.1037/0012-1649.43.5.1124 17723040

[B66] Scott L. S. Tanaka J. W. Sheinberg D. L. Curran T. (2008). The role of category learning in the acquisition and retention of perceptual expertise: a behavioral and neurophysiological study. *Brain Res.* 1210 204–215 10.1016/j.brainres.2008.02.054 18417106

[B67] Seitz A. R. Kim D. Watanabe T. (2009). Rewards evoke learning of unconsciously processed visual stimuli in adult humans. *Neuron* 61 700–707 10.1016/j.neuron.2009.01.016 19285467 PMC2683263

[B68] Serences J. T. Yantis S. (2006). Selective visual attention and perceptual coherence. *Trends Cogn. Sci.* 10 38–45 10.1016/j.tics.2005.11.008 16318922

[B69] Shafto P. Kemp C. Bonawitz E. B. Coley J. D. Tenenbaum J. B. (2008). Inductive reasoning about causally transmitted properties. *Cognition* 109 175–192 10.1016/j.cognition.2008.07.006 18952205

[B70] Shiffrin R. M. Schneider W. (1977). Controlled and automatic human information processing: II. Perceptual learning, automatic attending and a general theory. *Psychol. Rev.* 84 127 10.1037/0033-295X.84.2.127

[B71] Sloutsky V. M. (2010). From perceptual categories to concepts: what develops? *Cogn. Sci.* 34 1244–1286 10.1111/j.1551-6709.2010.01129.x 21116483 PMC2992352

[B72] Sloutsky V. M. Fisher A. V. (2008). Attentional learning and flexible induction: how mundane mechanisms give rise to smart behaviors. *Child Dev.* 79 639–651 10.1111/j.1467-8624.2008.01148.x 18489418

[B73] Sloutsky V. M. Kloos H. Fisher A. V. (2007). When looks are everything: appearance similarity versus kind information in early induction. *Psychol. Sci.* 18 179–185 10.1111/j.1467-9280.2007.01869.x 17425540

[B74] Smith L. B. Colunga E. Yoshida H. (2010). Knowledge as process: contextually cued attention and early word learning. *Cogn. Sci.* 34 1287–1314 10.1111/j.1551-6709.2010.01130.x 21116438 PMC2992382

[B75] Stanislaw H. Todorov N. (1999). Calculation of signal detection theory measures. *Behav. Res. Methods Instrum. Comput.* 31 137–149 10.3758/BF03207704 10495845

[B76] Treisman A. M. Gelade G. (1980). A feature-integration theory of attention. *Cogn. Psychol.* 12 97–136 10.1016/0010-0285(80)90005-5 7351125

[B77] Tversky A. (1977). Features of similarity. *Psychol. Rev.* 84 327–352 10.1037/0033-295X.84.4.327

[B78] Vossel S. Geng J. J. Fink G. R. (2014). Dorsal and ventral attention systems distinct neural circuits but collaborative roles. *Neuroscientist* 20 150–159 10.1177/1073858413494269 23835449 PMC4107817

[B79] Watanabe T. Sasaki Y. (2015). Perceptual learning: toward a comprehensive theory. *Annu. Rev. Psychol.* 66 197–221 10.1146/annurev-psych-010814-015214 25251494 PMC4286445

[B80] Weissman D. H. Prado J. (2012). Heightened activity in a key region of the ventral attention network is linked to reduced activity in a key region of the dorsal attention network during unexpected shifts of covert visual spatial attention. *Neuroimage* 61 798–804 10.1016/j.neuroimage.2012.03.032 22445785

