# Supplementary material for: Feature saliency and feedback information interactively impact visual category learning
Source: Front Psychol. 2015 Feb 19;6:74. doi: 10.3389/fpsyg.2015.00074 (PMC4333777; doi:10.3389/fpsyg.2015.00074)
Supplement: Supplementary file 1 [file Data_Sheet_1.DOCX]

**Appendix A: Stimuli**

**
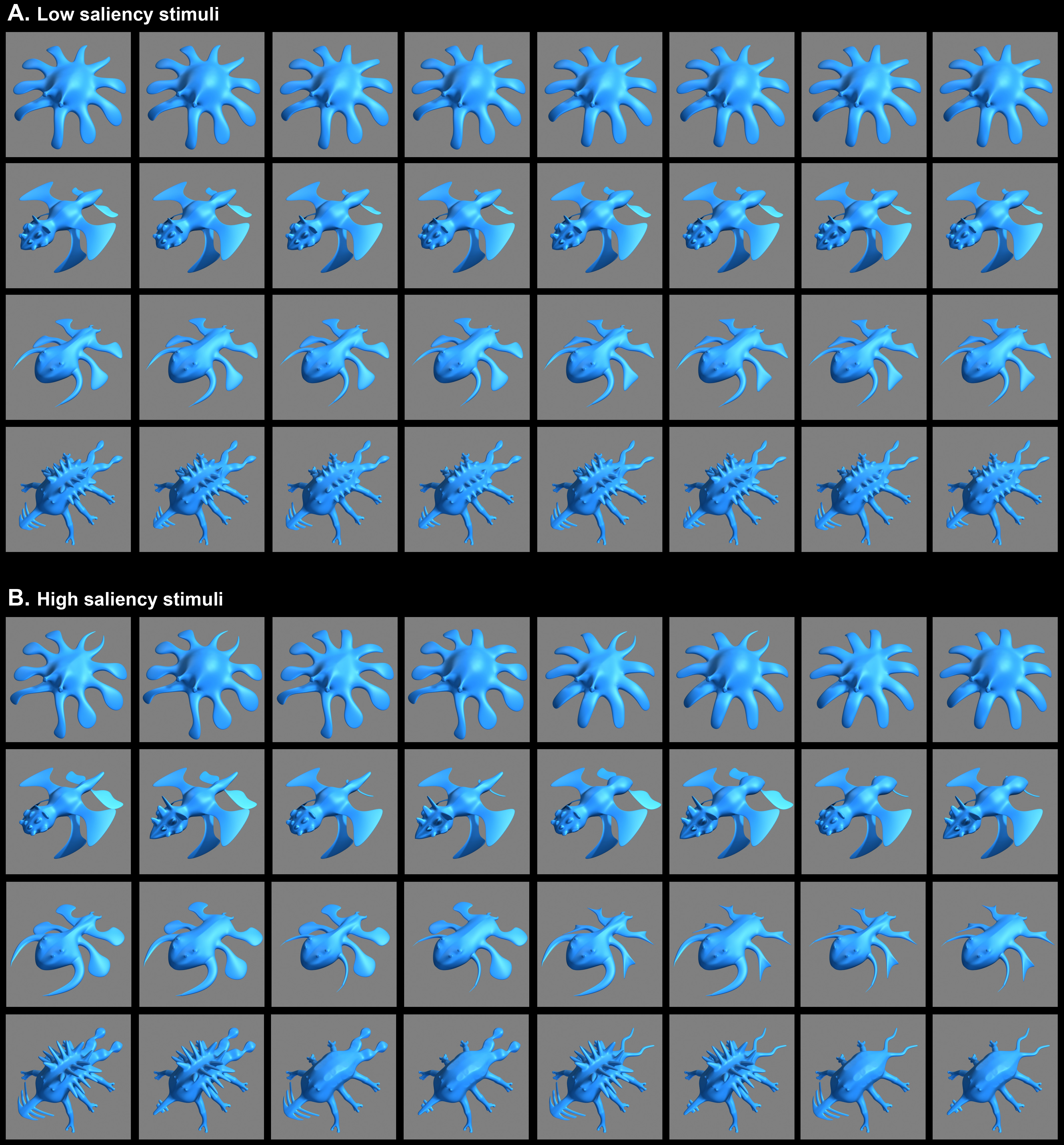
**

**Appendix B: Data exclusion criteria**

We excluded from the analysis visual category learning (VCL) tasks/runs in which participants did not respond to 25% or more trials, within one of the test or learning blocks (three cases: two in the low-saliency high-information feedback condition and one in the low-saliency mid-information feedback condition). In addition, in high-information VCL learning blocks, several participants did not follow the instructions and performed the task while reversing their responses (switching between the two response keys) while attending to an irrelevant feature dimension. This ‘strategy’ enabled participants to reach an apparent high performance level during the learning blocks but it was contrasted with poor performance in the test blocks. This resulted in the exclusion of 12 cases (VCL tasks) – eight in the low-saliency high-information feedback condition and four in the high-saliency high-information condition. Following these exclusions additional participants were tested so to keep the number of participants equal (n = 24) in all experimental conditions. These exclusion criteria were not applied to the unsupervised and no-information control conditions.

We used between-subjects statistical analyses for the between-conditions comparisons. This is due to the fact that no participant took part in all experimental conditions and thus within-subjects analyses are not applicable. The use of between-subjects statistical analyses under such conditions results in a more conservative statistical result with a lower likelihood for type-I error (Greenhouse & Geisser, 1959).
